# Foliar Application of Chitosan Increases Tomato Growth and Influences Mycorrhization and Expression of Endochitinase-Encoding Genes

**DOI:** 10.3390/ijms21020535

**Published:** 2020-01-14

**Authors:** Fatima El Amerany, Abdelilah Meddich, Said Wahbi, Andrea Porzel, Moha Taourirte, Mohammed Rhazi, Bettina Hause

**Affiliations:** 1 Natural Macromolecules Team, Normal Graduate School, Department of Biology, University Cadi Ayyad, PO Box 575, Marrakech 40000, Morocco; rhazimed@gmail.com; 2 Laboratory of Bio-Organic Chemistry and Macromolecular, Faculty of Science and Technology of Marrakech, Department of Chemistry, Cadi Ayyad University, PO Box 549, Marrakech 40000, Morocco; taourirte@gmail.com; 3 Laboratory of Biotechnology and Plant Physiology, Faculty of Science Semlalia Marrakech, Department of Biology, Cadi Ayyad University, PO Box 2390, Marrakech 40000, Morocco; a.meddich@uca.ma (A.M.); wahbi@ucam.ac.ma (S.W.); 4 Department of Bioorganic Chemistry, Leibniz Institute of Plant Biochemistry (IPB), Weinberg 3, 06120 Halle (Saale), Germany; Andrea.Porzel@ipb-halle.de; 5 Department of Cell and Metabolic Biology, Leibniz Institute of Plant Biochemistry (IPB), Weinberg 3, 06120 Halle (Saale), Germany; bhause@ipb-halle.de

**Keywords:** chitosan, arbuscular mycorrhizal fungi, tomato, chitosan nanoparticles, chitinase, gene expression

## Abstract

Nowadays, applying bio-organic fertilizer (e.g., chitosan, Ch) or integrating beneficial microorganisms (e.g., arbuscular mycorrhizal fungi, AMF) are among the successful strategies to promote plant growth. Here, the effect of two application modes of Ch (foliar spray or root treatment) and Ch-derived nanoparticles (NPs) on tomato plants colonized with the AMF *Rhizophagus irregularis* were analyzed, thereby focusing on plant biomass, flowering and mycorrhization. An increase of shoot biomass and flower number was observed in arbuscular mycorrhizal (AM) plants sprayed with Ch. The interaction with AMF, however, was reduced as shown by decreased mycorrhization rates and AM-specific gene expression. To get insights into Ch effect on mycorrhization, levels of sugars, jasmonates, abscisic acid, and the expression of two chitinase-encoding genes were determined in mycorrhizal roots. Ch had no effect on sugar and phytohormone levels, but the reduced mycorrhization was correlated with down- and upregulated expression of *Chi3* and *Chi9*, respectively. In contrast, application of NPs to leaves and Ch applied to the soil did not show any effect, neither on mycorrhization rate nor on growth of mycorrhizal plants. Concluding, Ch application to leaves enhanced plant growth and flowering and reduced interaction with AMF, whereas root treatment did not affect these parameters.

## 1. Introduction

Plant growth, characterized by cell division, cell expansion and changes in endogenous level of metabolites, gene expression, and tissues functions, is influenced by changes in the environment, such as changing supply of water, light, nutrients, and interactions of plants to other living organisms, leading often to alterations in levels of phytohormones that control plant responses.

In modern agriculture, there is a massive use of pesticides in order to promote growth and to prevent unacceptable losses of agricultural products. Although the application of these compounds has positive effects on plant growth and productivity, it often causes environment degradation and health risks. Recently, the application of natural products (e.g., Chitosan) attracted many attentions and is nowadays considered to be a very good alternative to pesticides [1,2].

Chitosan (Ch) is a carbohydrate derivative from chitin. Both of them are composed of D-glucosamine and N-acetyl-D-glucosamine units, which are linked by glycosidic bond β-(1→4). The increase in demand for Ch instead of chitin is due to its large function in different field areas [3]. The D-glucosamine unit, which is dominant in polymeric chain, increases the solubility of Ch in weak acidic solution [4]. Ch solutions are applied to produce biodegradable films, which are used in food preservation [5], to encapsulate drugs for delivery [6], and to disrupt the wall of microorganisms [7]. Recently, there are many studies focusing on the production of nanostructured Ch called nanoparticles (NPs) of Ch. The successful application of NPs is due to its nanometer size enabling uptake into plant cells, their large surface area, cationic nature, active functional groups, porosity and higher encapsulation efficiency [8].

Ch was found to promote growth in many plant species and its effects on plants needs in-depth research. As far as tomato (*Solanum lycopersicum* L.) cultivars concerned, most experiments were done to elucidate the enhanced protection of tomato against fungal attack and to reduce disease severity, e.g., caused by *Fusarium oxysporum, Fusarium andiyazi* [2,9], and *Alternaria solani* [10]. It has been shown that concentrations between 1mg/mL and 5mg/mL of Ch were highly effective against pathogenic fungi, supporting the assumption that its effect is due to putative physical barriers made around the plants that block the fungal invasion [9]. In addition, it has been shown that application of Ch increases the expression of genes encoding hydrolytic enzymes, such as β-1,3-glucanase and chitinases [2].

Besides its positive impact against pathogenic fungi, Ch might maintain the viability and might promote the activity of some beneficial micro-organisms, such as plant growth promoting rhizobacteria (PGPR) and arbuscular mycorrhizal fungi (AMF) [11,12]. As far as AMF are concerned, many studies showed that the mutualistic symbiosis between plants and AMF stimulated plant growth and promoted the root development through the increase of nutrient uptake (in particular P, [13]) and water retention [14]. Mycorrhization can also affect plant health either by increasing the tolerance to biotic and abiotic stresses or even by inducing disease resistance [15].

It is known that the association between AMF and plant is also regulated by hormones produced by the plant in a highly diverse manner [16]. Some of them trigger presymbiotic responses of the fungus (i.e., strigolactones), while others could initiate and involve in regulation of early events of fungal growth (i.e., auxin) [17]. Additionally, it was reported that the accumulation of jasmonate and abscisic acid could operate in symbiosis, in a dose dependent manner [18,19].

Even AMF are non-pathogenic fungi, an AM-induced expression of defense protein (PR-protein) genes, such as those encoding chitinase (class III), in roots of *Allium porrum* (leek) [20] and *Medicago truncatula* [21]. This class of chitinase appeared to be more closely linked to fungal enzymes involving in morphogenesis [22], suggesting its implication in the early events of symbiosis. However, the role of chitinase is marred with confusion due to its contribution in defense mechanisms against pathogens.

A recent study showed that AMF are able to take up recalcitrant (i.e., relatively large and complex) organic nitrogen and that Ch is localized in intraradical hyphae of AMF [23]. It has been speculated that mycorrhizal fungi process significant amounts of organic nitrogen in ecosystems, thereby improve plant N acquisition and ultimately plant productivity [23]. Thus, the combination of AMF with Ch may exert a cooperative effect on plant growth. However, there is only limited information about their mechanisms as well as the question, who will impact the other one first. In addition, it had been reported that the amendment of soil with Ch increased the colonization of tomato roots with AMF only in nutrient-deficient soil; however, the mycorrhization pathway was deactivated under the amendment of soil with combined bio-fertilizers: Ch and compost [24].

In this study, we evaluated the effect of Ch extracted from crustacean shrimps and its derived NPs on tomato growth. To get deeper insight into the interaction between chitinous products and AMF, as well as its effects, we have studied the effect of two application modes of Ch (foliar spray and soil drenching) and one application mode of NPs (foliar spray) in combination with the AMF *Rhizophagus irregularis* on tomato growth, mycorrhization rate and expression of genes encoding endochitinases.

## 2. Results

### 2.1. Degree of Deacetylation (DDA) of Ch and Morphology of NPs

**Figure 1:**
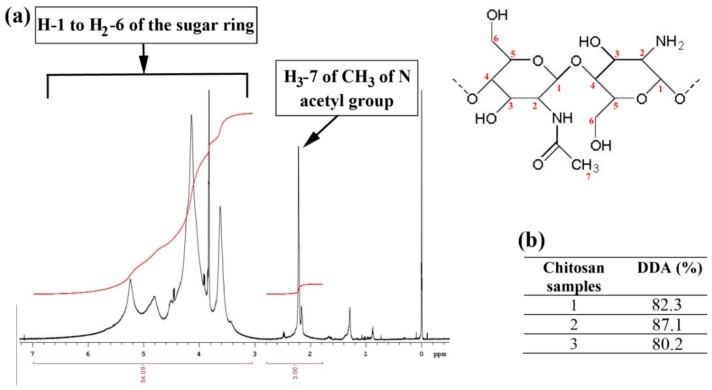
^1^H NMR spectrum of (**a**) solubilized chitosan in deuterated formic acid (HCOOD). (**b**) Degree of deacetylation (DDA) of three samples of chitosan.

To characterize the quality of chitosan produced, its DDA was determined by ^1^HNMR. The ^1^HNMR spectra (Figure 1a) showed a small peak area at 2.2 ppm which was assigned for the three protons of N acetyl group (-CO-CH_3_), and a large area of peaks between ca. 3.0 to 6.0 ppm that was attributed to the seven protons of glucose skeleton of Ch. The analysis of three samples of Ch (Figure 1b) by ^1^HNMR revealed that the DDA average of Ch was equal to 83%. It shows that Ch was not completely deacetylated.

**Figure 2:**
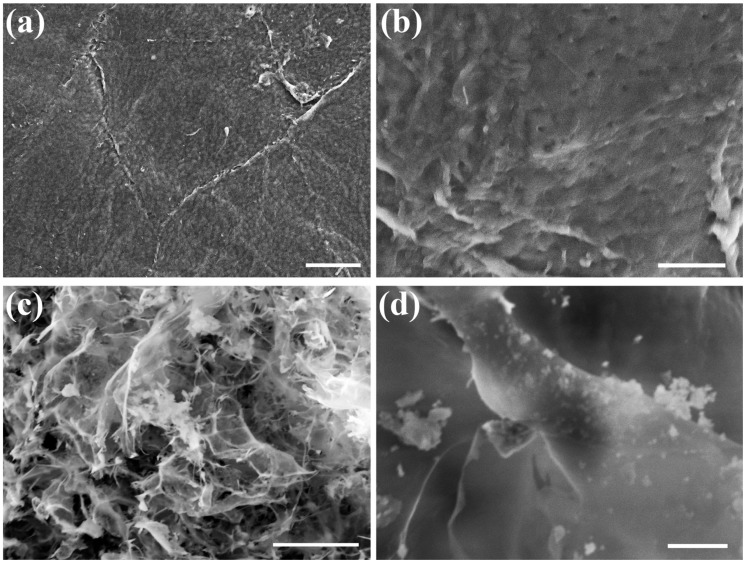
SEM image of chitosan (Ch, **a**,**b**) and chitosan nanoparticles (NPs, **c**,**d**). Bars represent 20 µm in (**a**,**c**) and 5 µm in (**b**,**d**).

Ch is treated with sodium tripolyphosphate (TPP) due to its ability to create five ionic cross-linking points with amino groups of Ch. Therefore, to show the changes that occurred during the addition of TPP to Ch, the surface structure of Ch and NPs was visualized by SEM (Figure 2). The analyses showed that Ch surface was homogeneous, compact and non-smooth with holes, which appeared to be not very deep (Figure 2a,b). However, the surface of Ch was changed completely after treatment with TPP resulting in a microscaffold structure (Figure 2c,d). This shape was characterized by different sized microfibers connected to each other and a higher porosity structure. In addition, nanoparticles were observed on the whole surface of microfibers.

### 2.2. Chitosan and Nanoparticles Effects on Tomato Growth and Mycorrhization

To analyze the effects of treatment with Ch or NPs on plant performance, non-mycorrhizal and mycorrhizal plants were treated every second week with different concentrations of Ch (0, 0.25, 0.50, 0.75 and 1.0 mg/mL) or NPs (0, 0.25, 0.50, 0.75 and 1.0% [*w*/*v*]). Thereby, Ch was applied in two different modes, either by spraying leaves or by soil drenching. After 45 d, shoot and root fresh weight, flowers number of mycorrhizal plants, and mycorrhization rate were determined.

**Figure 3:**
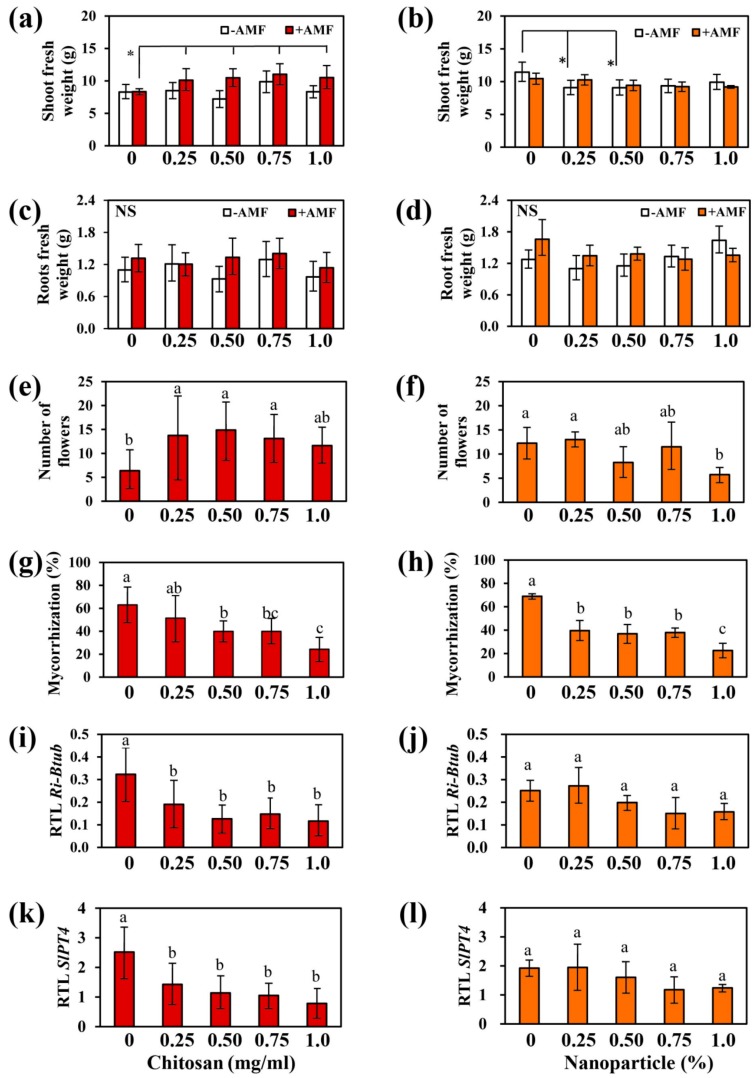
Effect of foliar spray with Ch and NPs on shoot (**a**,**b**) and root biomass (**c**,**d**) of plants inoculated or non-inoculated with *R. irregularis*, flowers number of inoculated plants (**e**,**f**), mycorrhization rate (**g**,**h**), and relative transcript levels of β-Tubulin (*RiBtub*) (**i**,**j**) and AM-specific phosphate transporter (*SlPT4*) genes (**k**,**l**). Data are represented as the mean ± SD with *n* =8 for Ch and *n* = 4 for NPs, different letters show significantly different values after one-way ANOVA followed by Duncan’s multiple range test (*p* ≤ 0.05).

Ch application to leaves had no effect on shoot and root growth of non-mycorrhizal plants. Similar results were obtained after application of NPs, except for shoot fresh weight that was decreased under the application of the lowest concentrations of NPs (0.25% and 0.50%) (Figure 3a–d).

Foliar spray of leaves with Ch increased, however, shoot fresh weight of mycorrhizal plants by about 21, 26, 32, and 26% at 0.25, 0.50, 0.75, and 1 mg/mL Ch, respectively, compared to non-treated plants (Figure 3a). In contrast, NPs application to shoots had no effect on growth of mycorrhizal plants (Figure 3b,d).

Furthermore, flower numbers of mycorrhizal plant were increased by foliar treatment with Ch. After application of medium concentrations of Ch, flower numbers were nearly doubled in comparison to non-treated plants (Figure 3e). In contrast, application of NPs did not affect significantly the flower number of mycorrhizal plants, except the highest concentration of 1% which decreased it to 50% (Figure 3f).

In comparison to non-treated plants, mycorrhization rate was significantly reduced in the range from 18 to 61% in plants treated with Ch (Figure 3g). Although there was no effect on the shoot biomass of mycorrhizal plants treated with NPs, the mycorrhization rate was also reduced from 42 up to 67% in plants treated with NPs (Figure 3h).

In order to confirm the data obtained by staining, levels of transcripts of genes encoding AMF marker genes were determined by RT-qPCR (Figure 3). β-Tubulin (*Ri-Btub*) and AM-specific phosphate transporter (*SlPT4*) genes were used as marker for fungal biomass and functional arbuscules, respectively [25,26]. Transcript levels of both genes were down regulated by application of Ch and NPs compared to non-treated plants (Figure 3i–l) supporting the fact that mycorrhization is indeed negatively affected by application of Ch and NPs to shoots.

**Figure 4:**
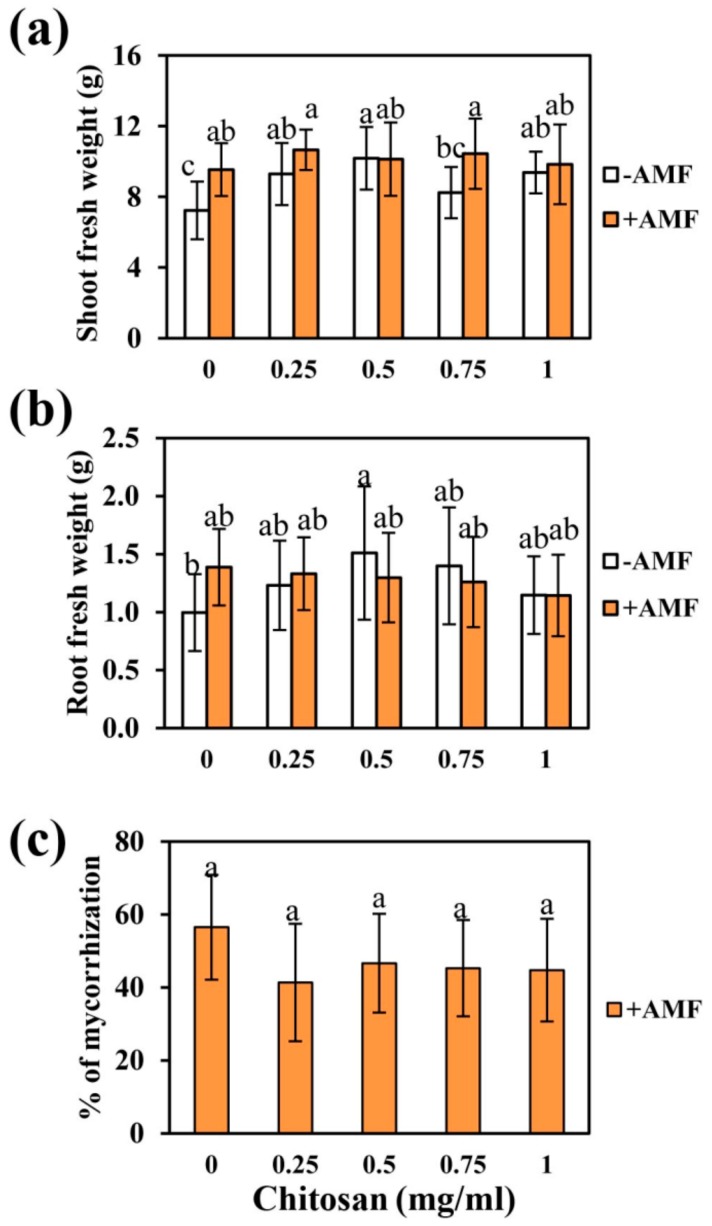
Effect of Ch root treatments on shoot (**a**) and root (**b**) biomass of plants inoculated and non-inoculated with *R. irregularis,* and on mycorrhization rate (**c**). Data are represented as the mean ± SD of eight biological replicates (*n* = 8), different letters show significantly different values after one- and two-way ANOVA followed by Duncan’s multiple range test (*p* ≤ 0.05).

After soil drenching with Ch, the shoot fresh weight was increased in treated plants in comparison to non-treated plants. Furthermore, the root growth was significantly increased in plants treated with the medium concentration of Ch (0.50 mg/mL). However, Ch application to plants inoculated with AMF did not lead to significant differences in the growth of shoots and roots (Figure 4). In addition, there was no alteration in the mycorrhization rate detected.

To get a deeper insight into the effect of foliar application of Ch on mycorrhization, contents of glucose, fructose, and, sucrose were determined in roots of mycorrhizal plants non-treated or treated with Ch by foliar application. The application of Ch; however, did not change significantly the level of all sugars in comparison to plants non-treated with Ch (Table 1).

### 2.3. Levels of ABA and Jasmonates, and Transcript Accumulation of ABA and JA-Related Genes During Mycorrhizal Symbiosis and Foliar Application of Ch

The effect of foliar spray with Ch on the level of jasmonates and ABA and their responsive genes was determined in roots of mycorrhizal and non-mycorrhizal plants at late stage of the symbiosis (nine weeks after inoculation).

**Figure 5:**
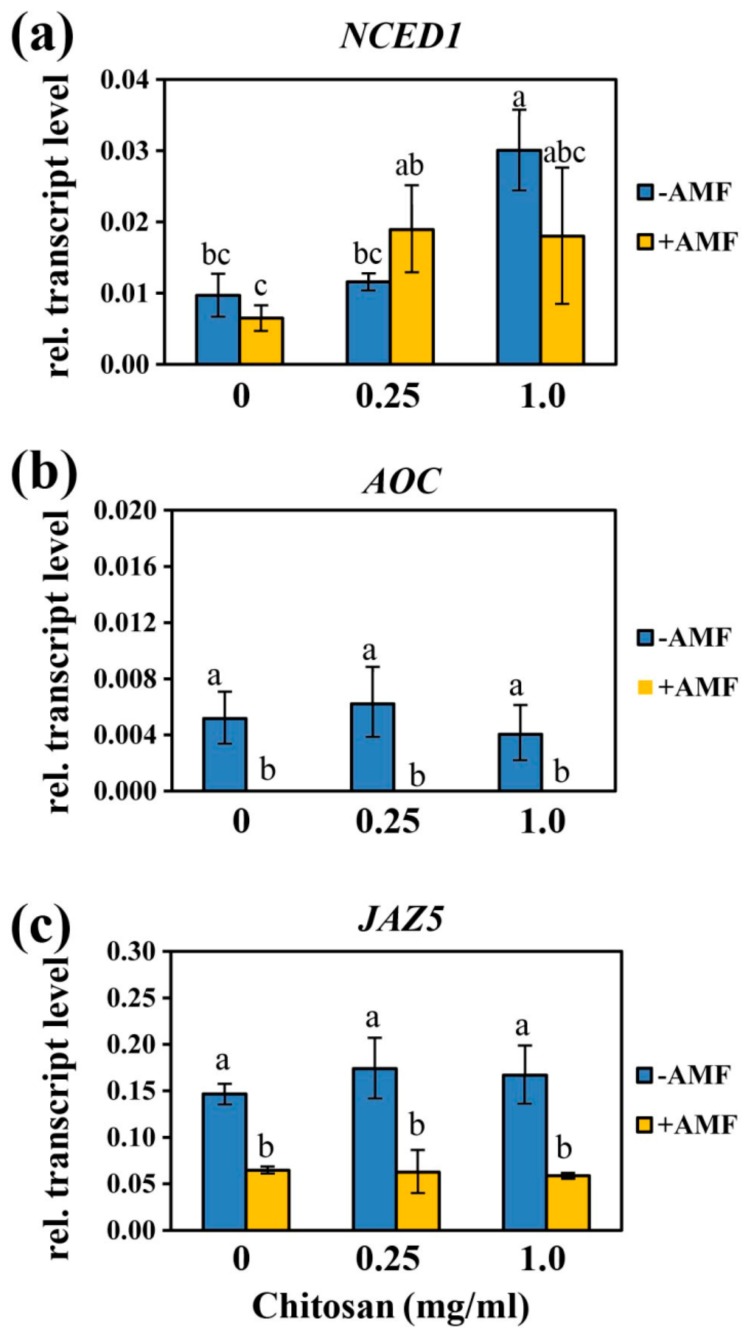
Effects of foliar spray with Ch on expression of genes encoding enzymes of ABA and JA biosynthesis or JA perception in roots inoculated and non-inoculated with *R. irregularis.* (**a**) 9-cis-epoxycarotenoid dioxygenase gene (*NCED1*), (**b**) allene oxide cyclase (*AOC*), (**c**) jasmonate-ZIM-domain protein 5 (*JAZ5*). Data are represented as the mean ± SD (*n* = 4), different letters show significantly different values after two-way ANOVA followed by Duncan’s multiple range test (*p* ≤ 0.05).

Quantification of ABA showed that treatment with Ch did not affect significantly the level of ABA in mycorrhizal and non-mycorrhizal roots (Table 2). However, the expression of 9-cis-epoxycarotenoid dioxygenase gene (*NCED1*), a gene encoding one of the ABA-biosynthetic enzymes [27], showed different results (Figure 5a). The expression of *NCED1* increased with increasing concentrations of Ch. The transcript accumulation of *NCED1* was significantly increased in roots of non-mycorrhizal plants treated with 1mg/mL of Ch.

Regarding jasmonates, levels of 12-oxo-phytodienoic acid (OPDA), jasmonic acid (JA) and jasmonoyl-isoleucine (JA-Ile) were determined. In comparison to non-mycorrhizal plants, the symbiotic interaction between *R. irregularis* and tomato roots resulted in a remarkable decline in the level of OPDA and JA with 52% and 77%, respectively (Table 2). Levels of JA-Ile, however, did not change significantly. Foliar spray with Ch did not affect the levels of OPDA, JA, and JA-Ile, neither in non-mycorrhizal nor in mycorrhizal roots.

To examine the effects of mycorrhization and treatment with Ch on genes expression of JA biosynthesis and signaling, transcript levels of allene oxide cyclase (*AOC*) and jasmonate-ZIM-domain protein 5 (*JAZ5*) were determined by RT-qPCR. *JAZ5* and *AOC* genes are among the genes that are highly expressed in roots during stress and mycorrhizal symbiosis which make them a targeted gene for this study [28,29,30]. The transcript level of these genes were down-regulated in mycorrhizal roots in comparison to non-mycorrhizal roots confirming the effect of AM on the levels of JA and OPDA (Figure 5b,c) for both types of roots. Ch application did not change the transcript levels of *AOC* and *JAZ5*.

### 2.4. Transcript Levels of Chitinase-Encoding Genes Changed Concomitantly to the Alteration of Mycorrhization

**Figure 6:**
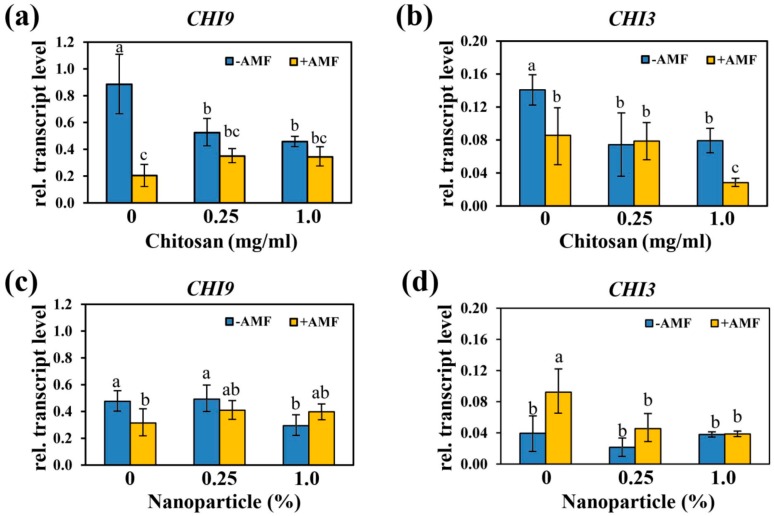
Effects of foliar spray with Ch (a and b) and NPs (c and d) on expression of defense related genes encoding chitinases in roots inoculated or non-inoculated with *R. irregularis*. (**a**,**c**) Basic isoform (*Chi9*), and (**b**,**d**) acidic isoform (*Chi3*) of chitinase. Data are represented as the mean ± SD (*n* = 4), different letters show significantly different values after two-way ANOVA followed by Duncan’s multiple range test (*p* ≤ 0.05).

Expression of defense-related marker genes that influence the growth of AMF, such as basic isoform (Chi9) and acidic isoform (Chi3) of chitosan were studied [31]. Relative levels of *Chi9* and *Chi3* transcripts in mycorrhizal and non-mycorrhizal roots of plants treated with Ch and NPs were determined by RT-qPCR (Figure 6). In non-treated plants, the transcript accumulation of *Chi9* in mycorrhizal roots was significantly reduced in comparison to non-mycorrhizal roots (Figure 6a,c), whereas transcript levels of *Chi3* showed only minor, but contrasting changes (Figure 6b,d). The application of highest concentration of Ch (1 mg/mL) to leaves significantly reduced the transcripts of *Chi9* in non-mycorrhizal plants; but, it did not affect their levels in mycorrhizal plants. In contrast, transcript levels of *Chi3* were strongly decreased in mycorrhizal roots after application of 1 mg/mL Ch (Figure 6b). Treatment with NPs; however, did not change significantly the transcript levels of *Chi3* and *Chi9*, but it resulted in the same tendency of alterations (Figure 6c,d).

In summary, the application of Ch and NPs resulted a reduction of the transcripts encoding the acidic isoform of chitinase, Chi3. This reduction correlates with the reduced mycorrhization after application of Ch and NPs to leaves of mycorrhizal plants (Figure 3g,h).

## 3. Discussion

Chitosan (Ch) extracted from crustacean shrimps and its derived nanoparticles (NPs) were applied to tomato plants and the effects of two application modes of Ch (foliar spray and soil drenching) in combination with mycorrhization by *R. irregularis* were evaluated. The data revealed that there was a promotion of plant growth accompanied with a decrease in mycorrhization, when Ch was applied to leaves, but application of NPs to shoots affected negatively the growth of plants and their mycorrhization. Moreover, there were no effects detectable after the application of Ch to soil. Ch and its precursor chitin applied to soil were often described as bio-fertilizer, thereby serving also as a source of carbohydrates to reinforce the mycorrhizal colonization in terms of improved colonization rate, increased length of extra-radical mycelium, and stimulated fungal spore formation [11].

Ch can interact with plant and elicit responses by two ways: via electrostatic interactions between their polycationic groups and plant’s polyanionic macromolecules, such as phospholipids, proteins, and lipopolysaccharides [32] and via interaction with chitin elicitor receptor kinase 1 (*CERK1*) [33,34]. The involvement of chitin receptor *CERK1* in perceiving chitosan signals is due to their sharing of N-acetylglucosamine (GlcNAc) unit. In the present study, treatment of AM roots with Ch, however, did not show an enhancement of neither plant growth nor mycorrhization rate. This might be due to the physical properties of Ch used. In fact, the amendment of soil with Ch has offer an excellent binding surface to nitrogen ions of compost [24] and its ability of chelating ions would be increased with its solubilization in a slight acidic solution leading to a protonation of amino groups. Therefore, the capacity of Ch to act as chelator might make some essential ions inaccessible to the plant [35].

In contrast to soil drenching, application of Ch to leaves of mycorrhizal plants had significant effects: Tomato plants treated with different concentrations of Ch showed enhanced shoot biomass, higher numbers of flowers and lower mycorrhization rates (Figure 3). El Amerany et al. [24] found similar result in tomato plant grown in soil amended with chitosan and compost. The positive effects of Ch on tomato growth and flowering might be due to its properties to act as anti-transpirant, to activate ROS scavenging system, to enhance stomatal conductance, and to stimulate growth of xylem vessels [24,36]. Moreover, application of Ch to leaves is known to increase the rate of photosynthesis leading to improved growth and overall plant development [37]. The reduction of mycorrhization was, however, rather unexpected. In the experiments presented here, tomato plants were grown under limited phosphate supply, thereby enforcing the interaction between AMF and plants [13]. Nevertheless, this mutualistic association can be negatively affected by certain conditions, such as competition with other microorganisms presented in the soil [38], limitation in carbohydrate supply due to limitations in photosynthesis [39], and the presence of elicitors [40].

**Figure 7:**
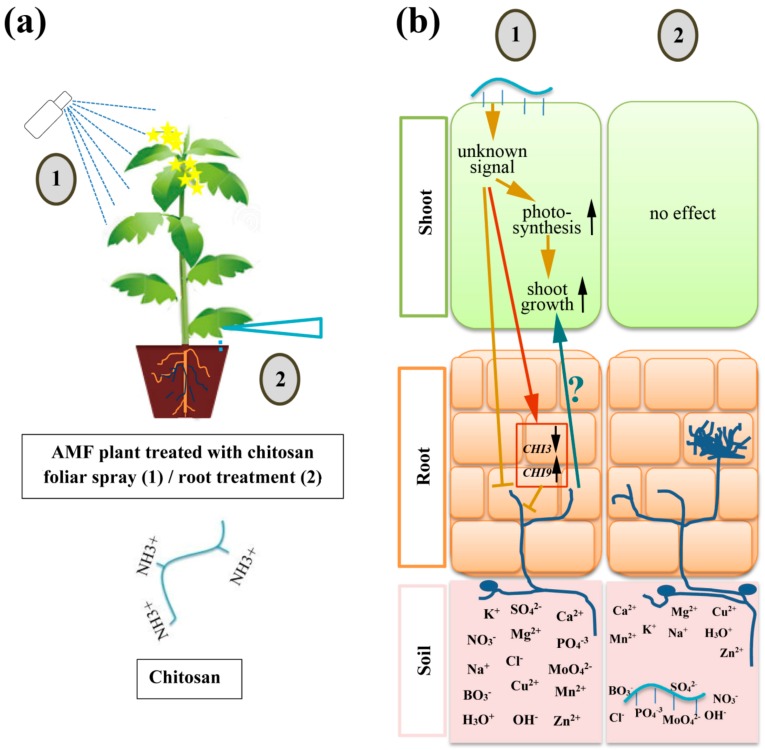
Model of the effect of two application modes of chitosan on mycorrhizal symbiosis between tomato and *R. irregularis*. (**a**) Two mode of applications of Ch were used in the experiments, foliar spray (1) and root treatment by soil drenching (2). (**b**) Putative mechanisms within the plant after application of Ch: (1) When chitosan is applied to shoot, it triggers some unknown signals that improve the photosynthesis followed by an increase in plant growth. In addition, foliar application of Ch leads to diminished mycorrhization, which could be related to changes in the expression of endochitinase genes (down regulation of *Chi3* and induction of *Chi9*), but could also contribute to enhanced plant growth. (2) The application of Ch to soil has no effect on growth and mycorrhization possibly due to its capacity to form complexes with soil ions.

Therefore, Ch application to the aerial part of plants might induce conditions that changed the interaction between plant and AM fungus. It is tempting to speculate that the increase in biomass and flower number limits the amount of carbohydrates delivered to the fungus. The limitation of sugar translocation from shoot to root tissues might block or at least reduce the symbiosis (Figure 7). To clarify whether application of Ch affects the levels of sugars in mycorrhizal roots, contents of glucose, fructose, and sucrose were analyzed. However, our results showed that the application of Ch did not change significantly the levels of all three sugars in roots of treated plants in comparison to roots of non-treated plants.

Other regulators of plant and mycorrhization are phytohormones, which play an important role in increasing plant growth and development and in establishment and functionality of the interaction between plant and AMF [16]. Moreover, there are other phytohormones regulating the plant immune responses, such as jasmonate and abscisic acid, whose role on mycorrhization have been thoroughly described [16,41,42]. Many studies showed that there is a correlation between increase levels of those hormones and mycorrhization rate [18,30].

In the presented work, the level of OPDA and JA were reduced upon mycorrhization; however, ABA levels did not change. These results could be explained by the fact that the level of JA and ABA may have been increased during early stages of the symbiosis and decreased when plant reached the advanced stage of mycorrhization (60–80%) at the end of the experiments, which has been shown also for *Phaseolus vulgaris* plants inoculated with *R. irregularis* for 4 weeks [43]. Additionally, the levels of OPDA, JA, JA-Ile, and ABA in mycorrhizal roots did not show any changes after treatment with Ch. These results were confirmed by the transcript level of genes involved in biosynthesis and signaling of JA (*AOC* and *JAZ5*, respectively) as well as ABA biosynthesis (*NCED1*). Therefore, alterations in the hormonal status of the mycorrhizal roots can be excluded as a reason for the diminished mycorrhization after Ch application.

Therefore, we speculated that the negative effect of Ch application on mycorrhization might be related to the activation of defense related genes. Genes encoding proteins involved in host defense responses, like chitinase and glucanase, are normally down-regulated, weakly or even not expressed during symbiotic interactions [21,44,45]. There are, however, genes encoding additional chitinase isoforms that belong to symbiosis-related genes, being specifically induced in response to the mycorrhizal association [46]. Plant genomes contain usually genes for five classes of chitinase, whereas one class (class III) is highly similar to microbial chitinases [47]. It has been speculated that the enzymes of class III plant chitinases are expressed during cell-wall morphogenesis and liberate chitin of micro-organisms (e.g., AMF) [20,22,48]. Regarding to symbiosis, class III plant chitinase could have another essential function as triggering plant immune system [49]. Thus, due to the lack of knowledge about the basic functions of class III and its effect on AM fungi during Ch application, we had suspected that the reduction of mycorrhization might be related to changes in the expression of two isoforms of chitinase-encoding genes of type III (*Chi3* and *Chi9*).

Regarding our data, we found that in response to treatment with Ch, both genes are regulated in a contrasting manner. *Chi9* showed an increase in transcript accumulation, whereas *Chi3* was down-regulated. The expression of basic and acidic chitinase-encoding genes with minimum and medium transcript, respectively, may be a mechanism to allow symbiosis with AM; however, the molecular mechanisms of *Chi3* on the growth of the fungi are still unknown. Furthermore, *Chi9* encodes a basic chitinase and its expression is up-regulated under conditions, where mycorrhization is reduced, such as growth of mycorrhizal plants in soil with a high phosphate content [50,51]. Moreover, this chitinase isoform seems to be involved in plant defense responses, since it is highly expressed in plants infected with the pathogenic fungus *Fusarium oxysporum* f. sp. *Lycopersici* [52].

Otherwise, Ch is also known to be a good elicitor of plant defense mechanisms [53]. The analysis of Ch samples by ^1^HNMR showed that Ch has 16% of N-acetylglucosamine units in its polymeric chain. This unit was considered as fingerprint that stimulates the expression of defense related genes. This experiment has provided a deeper insight about what’s happen to AMF during the application of Ch that is a source of exogene chitin oligomers, and coming up to the conclusion that those oligomers might be the reason of the increase of *Chi9* expression and the decrease of *Chi3* expression, leading to inhibition of AMF growth. Thus, the question that remains is how could foliar application alter chitinase gene expression in roots? Class III genes have been shown to be expressed either by hormones induction, such as salicylic acid and jasmonate, or by wounding [54,55]. Therefore, the analysis of phytohormones in shoot needs to be checked to close the gap in their implication in signal cross talk between shoot and root.

However, even AMF growth was affected, but their involvement in promoting plant growth was not excluded. In order to clarify the role of both chitinases, it would be interesting to see, how mutants affected in the expression of the respective genes will respond in respect to mycorrhization and application of Ch, and to get an overview about the effect of combined treatment (Ch + AMF) on plant growth as well as the failure of symbiosis network, transcriptomics analysis need to be done in future.

## 4. Materials and Methods

### 4.1. Chitosan and Nanoparticles Preparation

Chitin was isolated from shells of *Parapenaeus longirostris* which were subjected to strong acid and alkaline treatments as described by AL Sagheer [56]. Chitin was then deacetylated to produce Ch. Briefly, 1 g of chitin was stirred in 60% sodium hydroxide at room temperature for one d followed by incubation at 110 °C for 10 h. The resulting Ch was filtered, washed, and dried. Ch solution was prepared by dissolving 1 g of Ch in 1 L of 0.05% (*v*/*v*) acetic acid under constant stirring for one d. From this stock solution (1 mg/mL), various dilutions were prepared using 0.05% (*v*/*v*) acetic acid. The final pH of the solution was adjusted to 5.6 with NaOH.

NPs were prepared according to the protocol described by Dahmane [6]. Ch was dissolved in 1% (*v*/*v*) acetic acid and left under stirring to prepare 2.5 mg/mL of Ch solution. Then, 12 mL of Ch solution was added to 5 mL of 2.5 mg/mL aqueous sodium tripolyphosphate (Na_5_P_3_O_10_; TPP) by using a peristaltic pump to get a milky solution containing the produced NPs. NPs were sedimented by centrifugation at 10,000 rpm for 4 min and re-dispersed in deionized water. The stock solution of NPs was diluted with deionized water to prepare different concentrations of NPs.

### 4.2. Determination of Degree of Deacetylation of Chitosan by ^1^HNMR Spectroscopy

The DDA was calculated using three independently prepared samples of Ch, which were dissolved in deuterated formic acid (HCOOD) containing 1% of tetramethylsilane by stirring at room temperature for 48 h (Pharmacopeia; available online: https://www.drugfuture.com/Pharmacopoeia/USP35/data/v35300/usp35nf30s0_m2141.html, accessed on 10 July 2018).

The ^1^HNMR spectrum was obtained using HCOOD as solvent on an Agilent VNMRS 600 NMR spectrometer operating at a proton NMR frequency of 599.83 MHz using a 5 mm inverse detection cryo probe with the following parameters: Digital resolution, 0.367 Hz/point; pulse width (pw) = 6.5 μs (90°); relaxation delay = 27.3 s; acquisition time = 2.7 s; number of transients = 16.

For all spectra zero filling up to 128 K and an exponential window function with lb = 0.4 was used prior to Fourier transformation. ^1^H chemical shifts are referenced to internal tetramethylsilane (TMS) (δ ^1^H = 0 ppm).

DDA was determined using the following formula:

(1) DDA=100 1−7A23A1

A_1_ is the average area from about 3 ppm to 6 ppm that represents the seven protons of the sugar ring while, A_2_ is the average area of the signal at ca. 2.2 ppm, which represents the region of the proton next to carbonyl (CH_3_).

### 4.3. Analysis of Surface Morphology of NPs by Scanning Electron Microscopy (SEM)

To visualize the morphology of Ch and NPs, lyophilized Ch and NPs were loaded on grids, coated with graphite under vacuum using an automatic sputter coater and analyzed using a Scanning Electron Microscope (TESCAN VEGA 3).

### 4.4. Plant Materials and Treatments

Tomato (*Solanum lycopersicum* L., cv. MicroTom) seeds were germinated, planted in expanded clay of 2 mm to 5 mm particle size (Original Lamstedt Ton; Fibo Ex Clay, Lamstedt, Germany) and cultivated in a phytochamber with 16 h light (200 μmol photons m^−2^s^−1^) at 26 °C and 8 h dark at 20 °C, both at 70% humidity. Inoculation with *R. irregularis* was done by mixing expanded clay with inoculum harvested from mycorrhizal leek plants (0.85:0.15) according to Landgraf [57]. Plants were watered with distilled water and fertilized weekly with 10 mL of Long Ashton fertilizer containing 20% of phosphate of the original amount. After getting four leaves, plants were treated with Ch or NPs, which were applied every two weeks for a period of forty-five d. Deionized water was used as a control. Roots of mycorrhizal plants were harvested, weighted and flash frozen for RNA expression and hormone analysis while, a second part of roots was used directly after harvest for staining of AM structures. Eight replicates were used for Ch treatments, while four replicates were used for NPs treatments.

### 4.5. Sugar Measurement

The determination of sugar levels was carried out according to Schaarschmidt [58] using 50 mg of root fresh weight. Reactions were done in triplicates using glucose-6-phosphate dehydrogenase from *Leuconostoc mesenteroides* (Merck, Darmstadt, Germany) in a microplate reader (Sunrise) and recorded at the wavelength of 340 nm. Then, hexokinase (Merck) was added to determine the level of glucose followed by addition of phosphoglucose isomerase (Merck) and invertase (Merck) to determine levels of fructose and sucrose, respectively. Values were calculated using glucose, fructose, and sucrose as standards.

### 4.6. Phytohormone Measurement

OPDA, JA, JA-Ile, and ABA were quantified simultaneously using a standardized Ultra Performance Liquid Chromatography–tandem Mass Spectrometry (UPLC-MS/MS) based method according to Balcke [59]. In total, 50 mg of frozen plant material was extracted with methanol spiked with an appropriate amount of internal standards ([^2^H_5_]-OPDA, [^2^H_6_]-JA, [^2^H_2_]-JA-Ile, and [^2^H_6_]-ABA), pre-purified by solid phase extractions using a strong cation exchange HR-XC material (Macherey and Nagel, Düren, Germany) and finally subjected to LC-MS [59].

### 4.7. Determination of Mycorrhization Rate by Staining

Intraradical structures of *R. irregularis* were stained with black ink (Sheaffer, Providence, RI, USA) as described by Vierheilig [60]. Briefly, harvested roots were gently washed with water and the middle part of each root system (2–3 cm length) was dissected for staining. Root sections were incubated in 10% KOH at 90°C for 6 min followed by three washing steps with distilled water. Afterwards, the roots were incubated in 2% (*v*/*v*) acetic acid at room temperature for 10 min. Staining was performed using 5% (*v*/*v*) black ink in 2% (*v*/*v*) acetic acid. After incubation for 3 min at 90°C, samples were rinsed with water and stored at 4 °C for microscopic observation. Mycorrhization rate (M%) was determined using the gridline intersection method described by Giovanetti and Mosse [61].

### 4.8. RNA Extraction, cDNA Preparation and Real Time qPCR (RT-qPCR) Reaction

RNA isolation was done from 100 mg of homogenized root material using the RNeasy^®^ Plant Mini Kit (Qiagen, Hilden, Germany) according to the supplier’s protocol. Contaminating DNA was removed using DNA-free^Tm^ Kit (Thermo Fisher Scientific, Waltham, MA, USA). RNA concentration was determined using a NanoDrop 1000 spectrophotometer and the quality of RNA was checked on 1% (*w*/*v*) agarose gel and by PCR. cDNA was synthesized from 300 ng of RNA using the ProtoScript II First Strand cDNA Synthesis Kit (New England Biolabs GmbH, Frankfurt/M., Germany).

Transcript levels of eight genes were determined: Mycorrhiza marker genes (*Ri-βTub* and *SlPT4*), jasmonate-induced genes (*SlAOC* and *SlJAZ5*), ABA-induced gene (*SlNCED1*), defense related genes encoding chitinases (*Chi3* and *Chi9*) and a housekeeping gene (*SlEF*). For each reaction, 7 µl of master mix consisting of 2 µl EvaGreen (Bio&Sell, Feucht, Germany), 1 µl Forward Primer, 1 µl Reverse Primer (for primers see Table 3) and 3 µl water were mixed with 3 µl of cDNA (diluted 1:20) and the reaction was processed in a qPCR-machine CFX connect (Bio-Rad, München, Germany) using the following scheme: denaturation at 95 °C for 90 sec, incubation at 95 °C for 15 s and annealing for 30 s (the last two steps were repeated 39 times), final denaturation at 95 °C for 10 sec. At the end, a melting curve was recorded between 60 and 95 °C.

The data evaluation was done by the Bio-Rad CFX Manager and Excel 2010. The quantification cycle (Cq) value of the target gene (TG) was normalized to the reference gene (*SlEF*) and the relative transcript level (RTL) was calculated according to Schmittgen and Livak [62].

### 4.9. Statistical Analysis

All the data were analyzed by CoStat 6.400 (CoHort Software). The significance in differences between the treatments was determined using the analysis of variance (ANOVA) followed by Duncan’s test at *p* ≤ 0.05.

## 5. Conclusions

In summary, foliar spray with Ch had positive effects on biomass and number of flowers from mycorrhizal plants; but, it had a negative impact on the interaction with AMF. The reduced mycorrhization might be related to changes in the expression of chitinase genes (*Chi3* and *Chi9*). Moreover, application of Ch to leaves had stronger promoting effects on growth of mycorrhizal plants than application of NPs. Because of the smaller size of NPs, this result was not expected. Although NPs usually show a micro scaffold structure surrounded by different size of nanoparticles (this work, [63]), the application of NPs was not effective. The rather negative effect on plant growth is possibly due to the concentrations of NPs used. Therefore, further experiments will be required to adjust the concentration in order to get a positive impact on plant’s performance. Nevertheless, we can conclude that the application of Ch as foliar spray to mycorrhizal plants promotes growth and might replace chemical fertilizer.

## Figures and Tables

**Table 1:** Effects of foliar spray with Ch on glucose, fructose, and sucrose level in roots inoculated with *R. irregularis.* Data are represented as the mean ± SD of eight biological replicates (*n* = 8), different letters show significantly different values after one-way ANOVA followed by Duncan’s multiple range test (*p* ≤ 0.05).

Treatments ^1^	Glucose (mg/gFW)	Fructose (mg/gFW)	Sucrose (mg/gFW)
Ch_0_+AMF	31.07 ± 4.27 a	0.37 ± 0.11 a	77.55 ± 9.65 a
Ch_0.25_+AMF	27.91 ± 6.45 a	0.42 ± 0.13 a	68.71 ± 7.65 a
Ch_1_+AMF	30.11 ± 6.46 a	0.38 ± 0.10 a	71.96 ± 9.66 a

^1^ Ch_x_: different concentration of chitosan (0, 0.25 and 1 mg/mL); +AMF: inoculation with *R. irregularis.*

**Table 2:** Effects of foliar spray with Ch on levels of abscisic acid (ABA), 12-oxo-phytodienoic acid (OPDA), jasmonic acid (JA) and jasmonoyl-isoleucine (JA-Ile) in roots inoculated and non- inoculated with *R. irregularis.* Data are represented as the mean ± SD of eight biological replicates (*n* = 8), different letters show significantly different values after two-way ANOVA followed by Duncan’s multiple range test (*p* ≤ 0.05).

Treatments ^1^	ABA (pmol/gFW)	OPDA (pmol/gFW)	JA (pmol/gFW)	JA-Ile (pmol/gFW)
Ch_0_-AMF	43.83 ± 11.01 a	235.73 ± 43.06 a	258.88 ± 113.59 a	39.86 ± 11.23 ab
Ch_0.25_-AMF	46.42 ± 14.96 a	233.96 ± 41.14 a	176.91 ± 115.78ab	31.65 ± 4.44 b
Ch_1_-AMF	51.42 ± 10.83 a	278.97 ± 67.97 a	300.89 ± 136.62 a	52.05 ± 32.05 ab
Ch_0_+AMF	53.66 ± 9.34 a	112.49 ± 18.35 b	58.46 ± 15.12 b	58.48 ± 22.62 a
Ch_0.25_+AMF	53.35 ± 13.95 a	129.26 ± 13.82 b	64.27 ± 25.73 b	56.57 ± 29.78 a
Ch_1_+AMF	56.45 ± 11.16 a	131.69 ± 27.23 b	71.72 ± 33.80 b	39.56 ± 8.95 ab

^1^ Ch**_x_**: different concentration of chitosan (0, 0.25 and 1 mg/mL); -AMF: without inoculation with *R. irregularis;* +AMF: inoculation with *R. irregularis.*

**Table 3:** Primers used for RT-qPCR analysis.

Target Gene	Encoded Protein	Forward Primer	Reverse Primer
***Ri-βTub*** (MT007813)	β-Tubulin	CCAACTTATGGCGATCTCAACA	AAGACGTGGAAAAGGCACCA
***SlPT4*** (Solyc06g051850.1.1)	AM-specific Phosphate Transporter	TATGGCTGGATTTTGCTGCACGT	GAACTTGTATCATTCCCATCCGTC
***SlAOC*** (Solyc02g085730.2.1)	Allene Oxide Cyclase	TTCTACTTCGGCGATTACGGTC	GGTTAAGTACGCTCCCTGAACG
***SlJAZ5*** (Solyc03g118540)	Jasmonate ZIM-Domain5	CTATAACATCCCATGGTGGC	GAAGGAGATGGAAGAACTCC
***Chi3*** (Solyc02g082920)	Chitinase (Acidic Isoform)	TGCAGGAACATTCACTGGAG	TAACGTTGTGGCATGATGGT
***Chi9*** (Solyc10g055810.1.1)	Chitinase (Basic Isoform)	GAAATTGCTGCTTTCCTTGC	CTCCAATGGCTCTTCCACAT
***SlNCED1*** (Solyc07g056570)	9-Cis-Epoxycarotenoid Dioxygenase	ACATAATAGGCAAAGTCTCA	GTTGAAGAAGAAGAGGAGTT
***SlEF*** (Solyc11g069700.1.1)	Elongation Factor	ACCACGAAGCTCTCCAGGAG	CATTGAACCCAACATTGTCACC

## Abbreviations

ABA	Abscisic Acid
AMF	Arbuscular mycorrhizal fungi
AOC	Allene oxide cyclase
Ch	Chitosan
Chi	Chitinase
DDA	Degree of deacetylation
EF	Elongation factor
JA	Jasmonic acid
JA-Ile	Jasmonoyl-isoleucine
JAZ	Jasmonate-zim-domain protein
GlcNAc	N-acetylglucosamine
NCED1	9-cis-epoxycarotenoid dioxygenase
NPs	Nanoparticles of chitosan
OPDA	12-oxo-phytodienoic acid
SEM	Scanning electron microscopy
TPP	Sodium tripolyphosphate
